# A MAGEL2-deubiquitinase complex modulates the ubiquitination of circadian rhythm protein CRY1

**DOI:** 10.1371/journal.pone.0230874

**Published:** 2020-04-21

**Authors:** K. Vanessa Carias, Mercedes Zoeteman, Abigail Seewald, Matthea R. Sanderson, Jocelyn M. Bischof, Rachel Wevrick

**Affiliations:** Department of Medical Genetics, University of Alberta, Edmonton, AB, Canada; University of Lübeck, GERMANY

## Abstract

*MAGEL2* encodes the L2 member of the MAGE (melanoma antigen) protein family. Protein truncating mutations in *MAGEL2* cause Schaaf-Yang syndrome, and *MAGEL2* is one of a small set of genes deleted in Prader-Willi syndrome. Excessive daytime sleepiness, night-time or early morning waking, and narcoleptic symptoms are seen in people with Prader-Willi syndrome and Schaaf-Yang syndrome, while mice carrying a gene-targeted *Magel2* deletion have disrupted circadian rhythms. These phenotypes suggest that MAGEL2 is important for the robustness of the circadian rhythm. However, a cellular role for MAGEL2 has yet to be elucidated. MAGEL2 influences the ubiquitination of substrate proteins to target them for further modification or to alter their stability through proteasomal degradation pathways. Here, we characterized relationships among MAGEL2 and proteins that regulate circadian rhythm. The effect of MAGEL2 on the key circadian rhythm protein cryptochrome 1 (CRY1) was assessed using *in vivo* proximity labelling (BioID), immunofluorescence microscopy and ubiquitination assays. We demonstrate that MAGEL2 modulates the ubiquitination of CRY1. Further studies will clarify the cellular role MAGEL2 normally plays in circadian rhythm, in part through ubiquitination and regulation of stability of the CRY1 protein.

## Introduction

Prader-Willi Syndrome (PWS) is a genetic disorder of the nervous and endocrine systems characterized by developmental disabilities, hypotonia, hyperphagia, and obesity. Sleep apnea (obstructive and central), poor responses to hypoxia and hypercapnia, night wakening and narcoleptic symptoms contribute to abnormal sleep structure in individuals with PWS [1]. Excessive daytime sleepiness affects 90–100% of adults with PWS, according to parental reports [2]. Endocrine disruption, obesity and excessive daytime sleepiness are caused by hypothalamic dysfunction [3]. Therapies for excessive daytime sleepiness in PWS are largely focussed on relief of symptoms. For example, Modafinil was effective at reducing daytime sleepiness in an open label pilot study of children and adolescents with PWS, but has not yet been tested in a clinical trial setting [4].

Circadian rhythm is the oscillation of physiological and cellular functions, such as sleep and wake, over a 24-hour period. At a molecular level, the circadian clock functions through cell-autonomous transcriptional and post-translational feedback loops. The molecular clock is controlled by the synthesis, ubiquitination, and degradation of a key set of proteins [5]. A set of bHLH-PAS type transcription factors, including CLOCK, NPAS2, and BMAL1 heterodimerize and stimulate transcription from E-box containing promoters, including those driving period (*PER*) and cryptochrome (*CRY*) gene expression. PER and CRY proteins inhibit the CLOCK/BMAL1 complex, establishing a feedback loop. Cellular circadian rhythm relies on tightly regulated post-translational protein ubiquitination and proteasomal degradation, in particular the degradation of CRY and PER proteins by SCF (Skp1–cullin–F-box)-E3 ligase complexes [6, 7]. Consistent with the importance of protein stability in circadian rhythm, a recent genetic study suggested that insomnia is associated with variants in genes involved in ubiquitin-mediated proteolysis and in genes expressed in the brain, skeletal muscle, and adrenal glands [8].

Of the cluster of genes inactivated in individuals with PWS, only one gene, *MAGEL2*, has an expression profile and protein function consistent with a role in excessive daytime sleepiness and night waking in PWS. Mutations in the *MAGEL2* gene alone cause Schaaf-Yang syndrome, a disorder with overlapping phenotypes with PWS [9, 10]. As the number of people diagnosed with Schaaf-Yang syndrome is still small, sleep and circadian rhythm have not been formally investigated in these individuals. In mice, *Magel2* is highly expressed in the brain, specifically in the dorsal suprachiasmatic nucleus of the hypothalamus, overlapping with vasopressin positive neurons [11]. *Magel2* is also expressed in skeletal muscle and adrenal glands [12]. *MAGEL2* encodes a 1249 amino acid protein that is a member of “MAGE” (melanoma antigen) protein family. MAGE proteins interact with RING-zinc finger-type E3 ubiquitin ligases and ubiquitin-specific proteases (deubiquitinases) and influence the ubiquitination of substrate proteins [13]. Specifically, MAGEL2 interacts with TRIM27 and USP7 to modify the ubiquitination and stability of WASH1 [14], and interacts with RNF41 and USP8 to modify the ubiquitination and stability of the ESCRT-0 (Endosomal Sorting Complexes Required for Transport) complex [15]. MAGEL2 interacts with and modulates the stability and activity of circadian rhythm proteins, such as the transcription factors CLOCK and BMAL1 and the transcriptional repressor PER2 [16]. In the context of circadian rhythm, USP7 interacts with CRY1 and CRY2 and stabilizes these proteins through deubiquitination [17]. Other proteins, including RBX1 and its co-factors the SKP1, CUL1, and FBXL proteins, promote the ubiquitination and degradation of CRYs [18, 19]. Gene-targeted mice carrying a *Magel2* deletion have a circadian rhythm defect, and while they do entrain to 12:12 lighting conditions, they have anomalous activity in the light period in a background of reduced and fragmented total activity [11].

Given that MAGEL2 regulates protein ubiquitination in other cellular processes, we hypothesized that MAGEL2 could also regulate the activity of E3 ubiquitin ligases and deubiquitinases important for circadian rhythm. In this study, we identified relationships among MAGEL2, RBX1, USP7, and CRY1. MAGEL2 modulates the ubiquitination and stability of CRY1 and alters its nuclear-cytoplasmic distribution. We propose that disruption of circadian rhythm in people with Prader-Willi syndrome may be caused by MAGEL2-dependent deficiencies in the ubiquitin-dependent regulation of CRY1 levels.

## Results

### MAGEL2 has circadian expression and is highly expressed in the suprachiasmatic nucleus, the area of the brain that controls circadian rhythm

We examined the expression pattern of *Magel2* in the adult mouse brain using the Allen Brain Atlas [20]. *Magel2* is highly expressed in the hypothalamus, and most highly in the paraventricular and suprachiasmatic nuclei of the hypothalamus (S1A and S1A’ Fig). Using data from CircaDB, a database of circadian gene expression [21], we investigated *Magel2* expression in brain tissues. Magel2 was previously identified as one of the most circadian genes in the mouse suprachiasmatic nucleus of the hypothalamus [22](S1B Fig, mRNA profile), where peak *Magel2* expression (phase 14) precedes peak *Cry1* expression by ~3 hours (phase 17.4) (S1C and S1D Fig). In humans, *MAGEL2* expression also has a 24 hour period, with peak expression between midnight and the predawn hours in the nucleus accumbens (phase 17.2) and in amygdala (phase 20.4) respectively [23]. Notably, expression of *Magel2* is disrupted in mice carrying a *Clock* gene mutation, indicating that *Magel2* is a clock-controlled gene [24] (S1C Fig). However, these data only capture timing of peak mRNA, and the timing of expression of MAGEL2 protein is not known.

### MAGEL2 is in the vicinity of circadian rhythm protein CRY1

**Fig 1:**
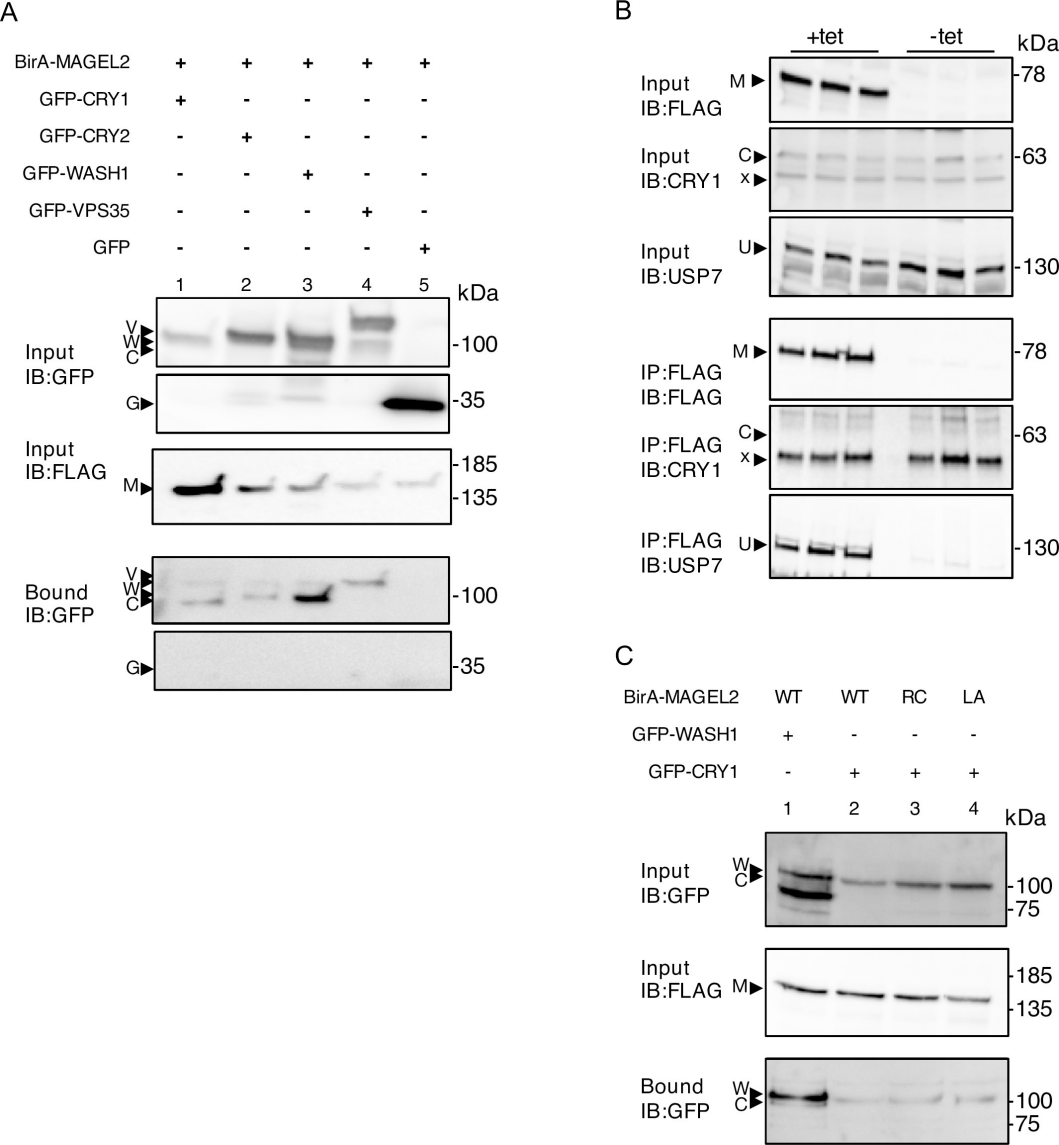
MAGEL2 is in proximity to CRY1 and CRY2 as detected by BioID. A) U2OS cells were transiently transfected with cDNA constructs encoding epitope-tagged proteins and incubated with excess biotin. After 24 h, cell lysates were collected, an aliquot was retained as input, and the remaining sample was processed by streptavidin affinity purification to recover proteins biotinylated by BirA* fusion proteins (bound). Input and bound samples were immunoblotted (IB) to detect recombinant proteins. BirA*-MAGEL2 (M) was co-transfected with GFP-tagged CRY1 (C) or CRY2 (C), or with GFP-WASH1 (W) or GFP-VPS35 (V) as interacting positive controls. GFP alone was used as a non-interacting negative control (G). B) FLAG-MAGEL2 (M) expression was induced (+tet), or not induced (-tet) with tetracycline in stably transfected HEK cells, then a co-immunoprecipitation experiment was performed, in triplicate, to detect interactions between FLAG-MAGEL2 and endogenous USP7 (U) or endogenous CRY1 (C). A cross-reacting protein is detected with the anti-CRY1 antibody at a smaller molecular weight than the endogenous CRY1 protein (x). C) BirA*-MAGEL2 (wildtype, WT), BirA*-MAGEL2p.R1187C (RC), or BirA*-MAGEL2p.LL1031AA (LA) were co-transfected with GFP-CRY1 or GFP-WASH1 (positive control) and processed for BioID.

We tested whether MAGEL2 interacts with the cryptochrome proteins CRY1 and CRY2, which are light-independent inhibitors of CLOCK:BMAL1 [17, 25]. We performed proximity-dependent biotin identification (BioID) in cells transiently co-transfected with a “bait” FLAG-tagged biotin ligase-fusion protein (BirA*-MAGEL2), and a second epitope-tagged protein as prey. After streptavidin-affinity purification of the cell lysate, biotinylated proteins were recovered and immunoblotted to detect proteins in proximity to the BirA*-fusion baits *in vivo* (bound). BirA*-MAGEL2 biotinylated GFP-CRY1 and GFP-CRY2 as demonstrated by their presence both in the input fraction and in the biotinylated proteins recovered by streptavidin affinity purification, indicating that MAGEL2 is in proximity (within ∼10 nm [26]) to CRY proteins (bound, Fig 1A, lanes 1, 2). As a positive control, BirA*-MAGEL2 also biotinylated GFP-WASH1 and GFP-VPS35, consistent with their interaction as detected previously by immunoprecipitation and mass spectrometry [27] (lanes 3 and 4). However, BirA*-MAGEL2 did not biotinylate GFP alone (lane 5, GFP present in input but not in bound fraction). Next, we performed a co-immunoprecipitation to examine the relationship between MAGEL2 and CRY1. While endogenous USP7 co-immunoprecipitated with FLAG-MAGEL2 in a stable cell line induced with tetracycline to express FLAG-MAGEL2, endogenous CRY1 did not co-immunoprecipitate with FLAG-MAGEL2 (Fig 1B). This confirms that USP7 interacts with MAGEL2, but suggests that while CRY1 is in the vicinity (within 10 nm) of MAGEL2, that these two proteins do not directly interact (Fig 1B). We then tested whether mutations that alter the MAGE homology domain (MHD) of MAGEL2 change the proximity of MAGEL2 to CRY1. The MHD of MAGE proteins interacts with E3 ubiquitin ligases, to variable regions outside of the active site of the E3 ligase protein [28], and these mutations could potentially affect the proximity of MAGEL2 to the substrate CRY1. BirA*-MAGEL2p.R1187C and BirA*-MAGEL2p.LL1031AA carry mutations that disrupt the MHD [15]. GFP-CRY1 was transfected along with either BirA*-MAGEL2p.R1187C or MAGEL2p.LL1031AA, which carry mutations in the MHD (Fig 1C). After transient transfection and BioID, wild-type BirA*-MAGEL2 biotinylated CRY1 as demonstrated by the presence of CRY1 in both in the input fraction and in the biotinylated proteins recovered by streptavidin affinity purification (bound, Fig 1C, lane 2). GFP-CRY1 was also recovered after BioID with BirA*-MAGEL2p.R1187C and BirA*-MAGEL2p.LL1031AA (Fig 1C, lanes 3, 4), suggesting that MAGEL2 and its mutant forms are all in proximity to CRY1.

### MAGEL2 co-expression affects CRY1 subcellular localization

**Fig 2:**
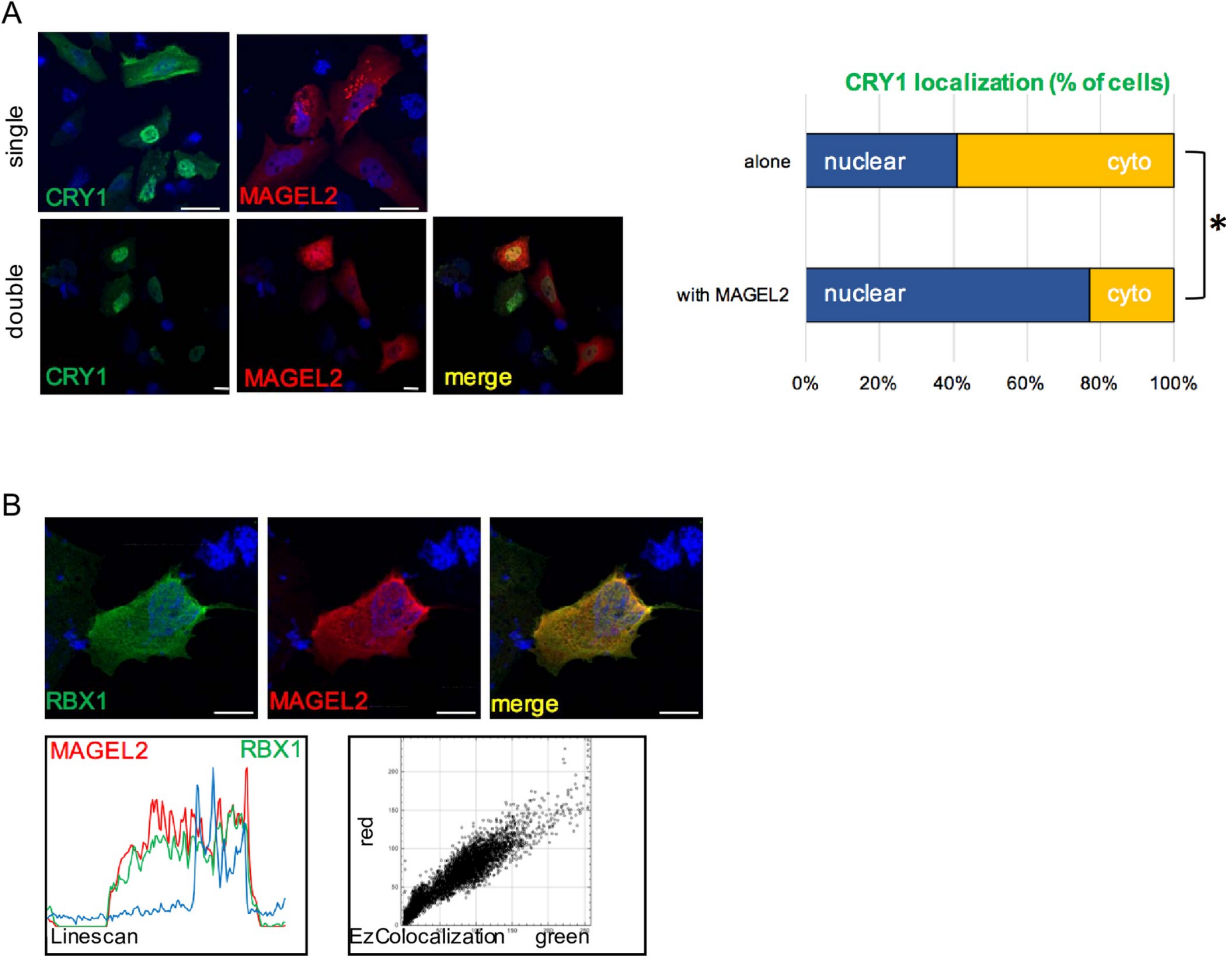
MAGEL2 modulates the subcellular localization of CRY1, and MAGEL2 co-localizes with RBX1 in the cytoplasm in transfected U2OS cells, A) Recombinant FLAG-CRY1 (green) or recombinant V5-MAGEL2 (red) either one at a time (single) or co-transfected (double) were detected in transfected U2OS cells. Nuclei are counterstained blue with Hoechst dye. Representative cells are shown. Scale bar, 10 μm. Difference between subcellular localization of CRY1 with and without MAGEL2, *P<0.0001, Fisher exact test, representative data from 1 of 3 experiments. B) Recombinant FLAG-RBX1 (green) and V5-MAGEL2 (red) were detected in co-transfected U2OS cells by immunofluorescence microscopy. Yellow signal in merged image indicates where protein expression overlaps in the cell. Examples of co-localization visualization (using Linescan in ImageJ) and analysis (using EzColocalization in ImageJ) are shown below the example cell image. Nuclei were counterstained blue with Hoechst dye.

MAGE proteins can influence the subcellular localization of E3 ligase-substrate complexes that they interact with [15], and the translocation of CRY to the nucleus is essential for its ability to suppress CLOCK:BMAL1–dependent transcription. We investigated whether MAGEL2 co-localizes with circadian rhythm proteins or affects their subcellular localization. MAGEL2 localized primarily to the cytoplasm when transfected into U2OS cells, while CRY1 is present in both the cytoplasm and the nucleus on transfection (Fig 2A). Notably, a higher proportion of cells had a nuclear CRY1 signal when MAGEL2 was co-expressed, compared to when CRY1 was transfected alone (example in Fig 2A). To quantify this effect, we compared the proportion of transfected cells in which the CRY1 was mostly in the nucleus to cells in which CRY1 was mostly in the cytoplasm, in cells transfected with the CRY1 construct alone or co-transfected with MAGEL2. While CRY1 was mostly nuclear in 42 of 102 (41%) of cells when transfected alone, CRY1 was mostly nuclear in 41 of 53 cells (77%) of cells when co-transfected with MAGEL2 (*P*<0.0001, Fisher exact test). However, we were unable to confirm an effect of MAGEL2 co-expression on the subcellular distribution of endogenous or exogenously expressed CRY1 in cultured cells using subcellular fractionation followed by immunoblotting (S2 Fig).

### A MAGEL2 interaction with RBX1 depends on the integrity of the MAGE homology domain

**Fig 3:**
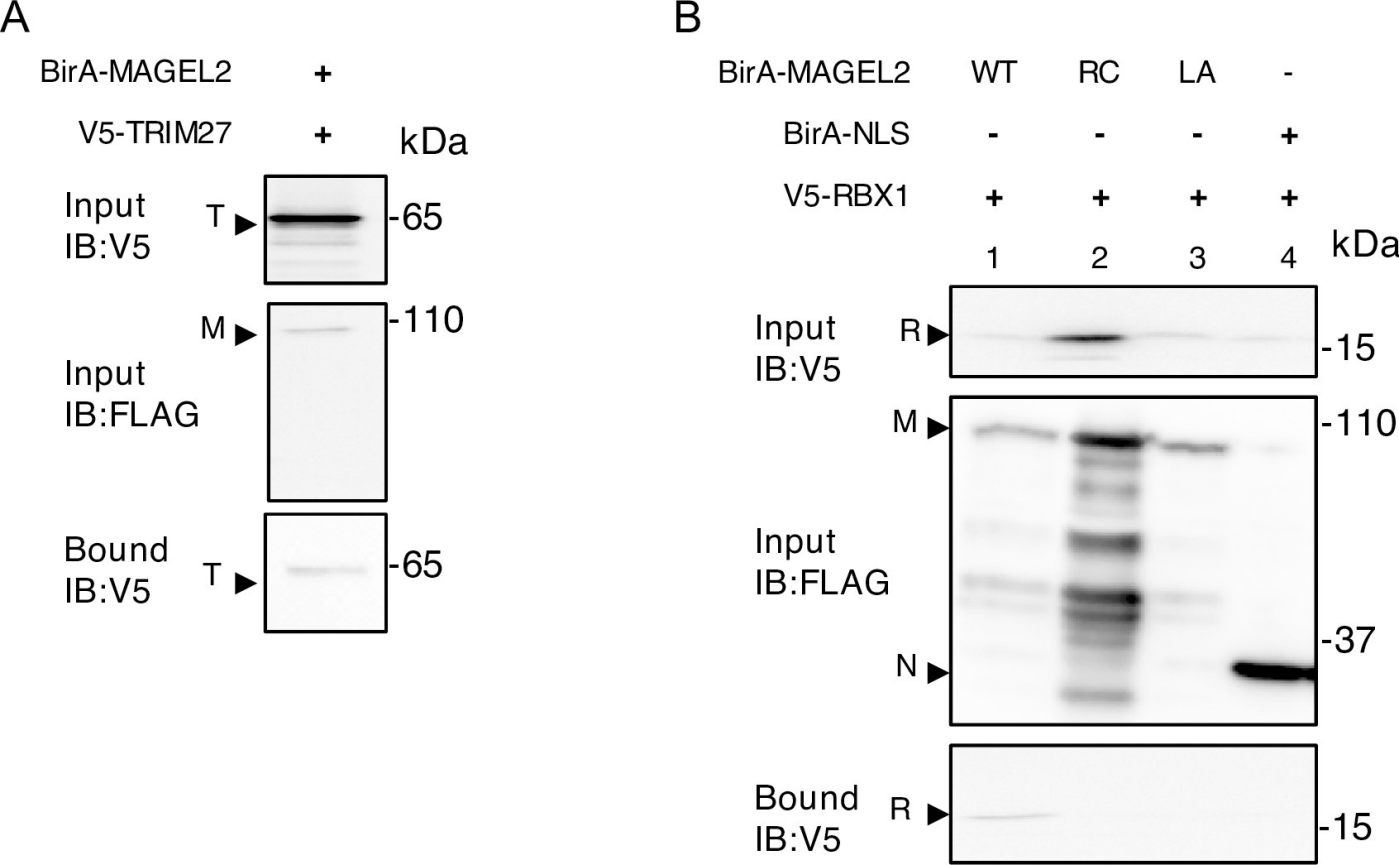
MAGEL2 is in proximity to RBX1 as detected by BioID. U2OS cells were transiently transfected with cDNA constructs encoding epitope-tagged proteins and incubated with excess biotin. After 24 h, cell lysates were collected, an aliquot was retained as input, and the remaining sample was processed by streptavidin affinity purification to recover proteins biotinylated by BirA* fusion proteins (bound). Input and bound samples were immunoblotted (IB) to detect recombinant proteins. A) BirA*-MAGEL2 was co-transfected with V5-tagged TRIM27 (T). B) BirA*-MAGEL2 (lane 1, wildtype, WT), BirA*-MAGEL2p.R1187C (lane 2, RC), BirA*-MAGEL2p.LL1031AA (lane 3, LA) or BirA*-NLS (N, lane 4) were co-transfected with V5-tagged RBX1 (R).

As MAGEL2 interacts with at least two E3 ubiquitin ligase complexes [14, 15], we hypothesized that MAGEL2 could also interact with RBX1-containing complexes, possibly including one or more of the SCF-E3 ligase complexes involved in circadian rhythm. MAGEL2 and RBX1 signals have some overlap in their localization in the cytoplasm in co-transfected cells (Fig 2B, yellow signal in the merged image, average overlap score of ~0.96, where 1 indicates complete overlap). Co-localization was visualized using Linescan (representative Linescan in Fig 2B) and quantified using EZColocalization, also in ImageJ. We then performed BioID in cells transiently co-transfected with a BirA*-MAGEL2 and RBX1. First, we confirmed that BirA*-MAGEL2 can biotinylate the E3 ligase TRIM27 (Fig 3A). BirA*-MAGEL2 also biotinylated V5-RBX1, indicating that RBX1 is in proximity (within ∼10 nm [26]) to MAGEL2 (Fig 3B). We then tested whether mutations that alter the MHD of MAGEL2 impair the ability of MAGEL2 to interact with RBX1. Unlike wildtype (WT) BirA*-MAGEL2, RBX1 was not biotinylated by BirA*-MAGEL2p.R1187C or BirA*-MAGEL2p.LL1031AA (Fig 3B, lanes 2 and 3). As a control, BirA*-NLS, which carries the biotin ligase cDNA fused to a nuclear localization signal (NLS), also did not biotinylate V5-RBX1 (lane 4).

### MAGEL2 promotes the ubiquitination of CRY1 and decreases CRY1 protein levels, opposing the effects of its interaction partner USP7

**Fig 4:**
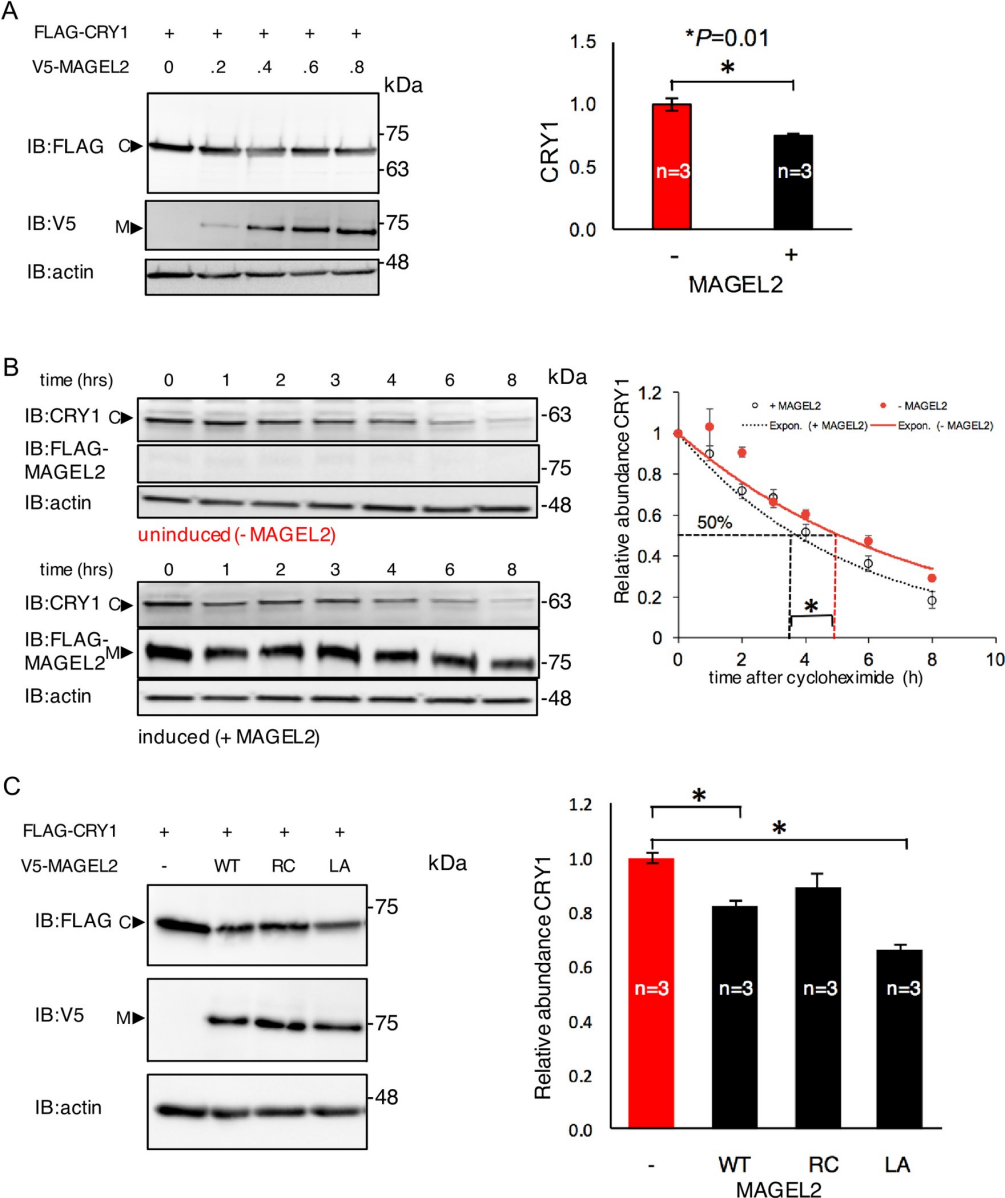
Co-expression of MAGEL2 reduces the abundance of CRY1. A) U2OS cells were transiently transfected with cDNA constructs encoding FLAG-CRY1 and varying amounts of V5-MAGEL2, and signal intensities were measured in cell lysates and normalized to signal intensity of actin (representative blot shown). Graph represents relative CRY1 signal intensity from a triplicate co-transfection at a 1:1 ratio of MAGEL2 (+) or empty vector (-) and FLAG-CRY1. *, *P* = 0.01 comparing empty vector to co-expression of MAGEL2. B) Stably transfected HEK cells were induced with tetracycline to express FLAG-MAGEL2 (+MAGEL2) or not (-MAGEL2) then treated with cycloheximide to inhibit new protein synthesis. The abundance of endogenous CRY1 was measured, normalized to actin abundance and to levels at time zero, then plotted *vs*. time after addition of cycloheximide to the cell culture (performed in triplicate, mean±SD, difference in half-life, *P<0.05). C) FLAG-CRY1 was co-transfected with V5-MAGEL2 wild-type (WT), V5-MAGEL2p.R1187C (RC), or V5-MAGEL2p.LL1031AA (LA) and the signal intensity of FLAG-CRY1 was measured in cell lysates (representative blot shown). Graph represents relative CRY1 signal intensity from a triplicate co-transfection at a 1:1 ratio of MAGEL2 (WT, RC, or LA or empty vector) and FLAG-CRY1. *, *P* = 0.01 comparing empty vector to co-expression of MAGEL2 (WT, RC, or LA).

MAGEL2 can affect protein stability and abundance through its role as a modulator of ubiquitination, and CRY1 abundance is regulated by SCF-E3 ubiquitin ligase complexes. Having established that MAGEL2 and CRY1 are transiently or lastingly in proximity to each other in cultured cells, we tested whether MAGEL2 could affect the abundance of CRY1. Cells were transfected with constructs expressing FLAG-CRY1 and varying amounts of V5-MAGEL2, with an empty vector used to equalize the amount of plasmid in all transfections (Fig 4A). Immunoblots of cell lysates demonstrated that the abundance of CRY1 protein decreased as MAGEL2 increased (Fig 4A). Measurements of CRY1 protein in lysates from cells singly or co-transfected with CRY1 and MAGEL2, in triplicate, demonstrated that co-expression of MAGEL2 reduced the amount of CRY1 detected by 25% compared to cells singly transfected with CRY1. We used a time course of cycloheximide treatment to measure the half-life of endogenous CRY1 protein in HEK cells that stably express a tetracycline-inducible FLAG-MAGEL2 construct. Consistent with the co-transfection results, MAGEL2 expression reduced the half-life of endogenous CRY1 protein 1.4-fold in HEK cells induced to express MAGEL2 compared to uninduced (3.7±0.06 h compared to 5.1±0.4 h, *P* = 0.03 by Student t-test, n = 3 replicates). We next tested whether MHD mutations affect the ability of MAGEL2 to alter CRY1 levels. While the wild-type MAGEL2 and MAGEL2p.LL1031AA decreased CRY1 protein levels, MAGEL2p.R1187C had no effect on CRY1 protein levels (data from triplicate transfections, example in Fig 4C). However, we did not detect any changes in Cry1 abundance in tissue from the brain (cortex or hypothalamus) of mice carrying a gene-targeted mutation in *Magel2* compared to analogous tissue from wild-type littermates (S3 Fig).

**Fig 5:**
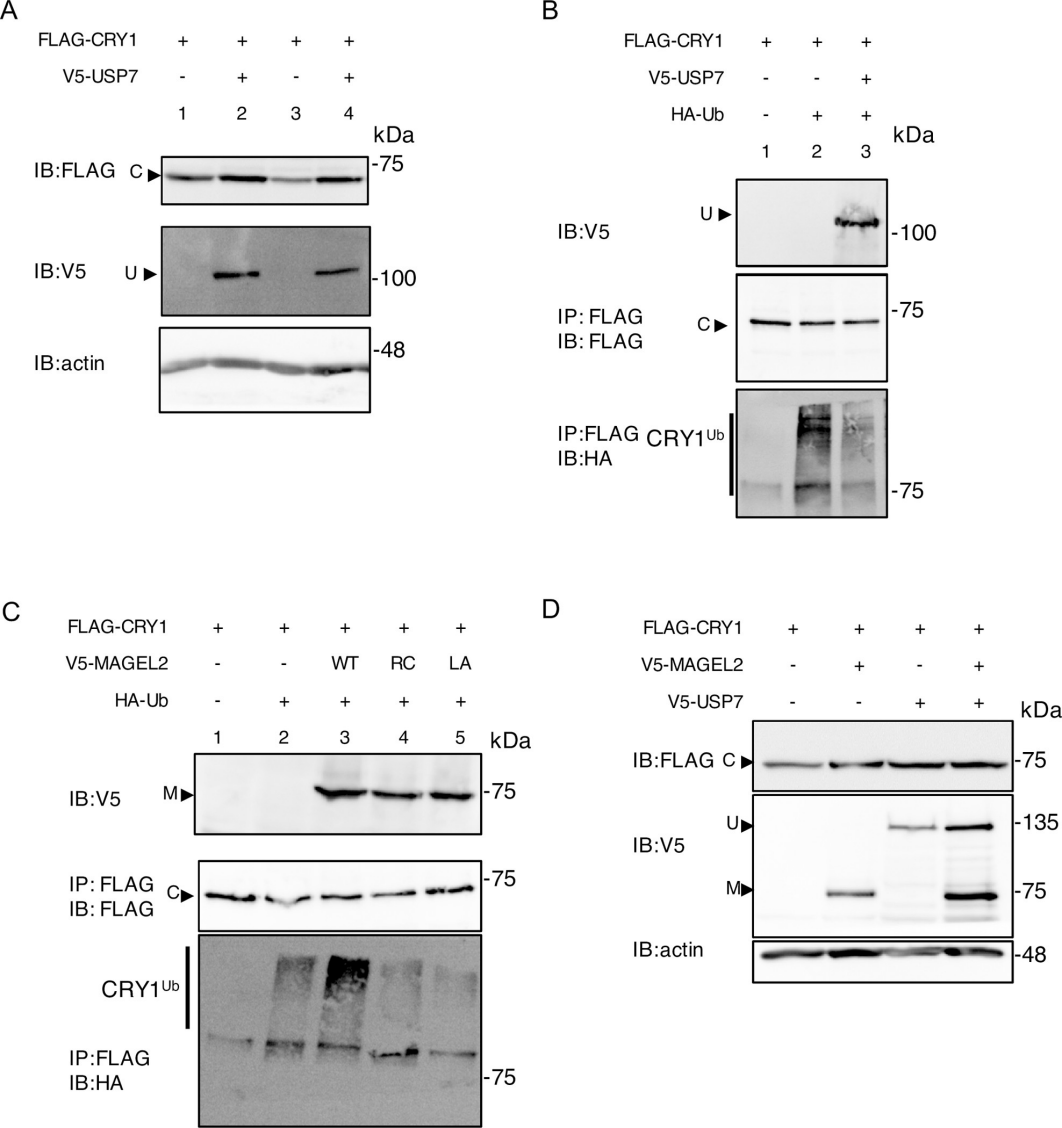
MAGEL2 and USP7 alter CRY1 stability through ubiquitination-related processes. A) USP7 stabilizes CRY1 when co-expressed in U2OS cells. U2OS cells were transiently transfected with cDNA constructs encoding V5-USP7 and FLAG-CRY1 in equal amounts. B) In a ubiquitination assay, HEK293T cells were co-transfected with epitope-tagged constructs and treated with MG132 and chloroquine. After 24 h, cell lysates were collected, and an aliquot was retained as input and immunoblotted (IB) to confirm expression of V5-USP7. FLAG-CRY1 was immunoprecipitated (IP) using anti-FLAG beads from the remaining lysate. FLAG-CRY1 was detected in the immunoprecipitate, and ubiquitinated CRY1 (Cry1^Ub^) was detected by probing the immunoprecipitate with anti-HA antibodies to detect HA-ubiquitin (HA-Ub, smear above molecular weight of CRY1). C) Ubiquitination assay showing effect of co-expression of MAGEL2 (V5-MAGEL2 wild-type (WT), V5-MAGEL2p.R1187C (RC), or V5- MAGEL2p.LL1031AA (LA)) on the ubiquitination of CRY1 (Cry1^Ub^) in transfected HEK cells. D) MAGEL2 and USP7 have opposing effects on the steady-state abundance of CRY1 in co-transfected cells.

While CRY1 is subject to ubiquitination by SCF-E3 ubiquitin ligase complexes, it is deubiquitinated and stabilized by USP7 [25]. Interactions between MAGEL2 and USP7 have been demonstrated by BioID [15]), and endogenous USP7 was recovered in an immunoprecipitation of stably expressed TAP-tagged MAGEL2 in HEK293 cells [27] and by co-immunoprecipitation with stably expressed FLAG-MAGEL2 (Fig 1B). We confirmed this interaction, performing a BioID experiment in U2OS cells transiently co-transfected with BirA*-USP7 as bait and MAGEL2 as prey (S4 Fig). Next, we examined the stability and ubiquitination of CRY1 in the presence of USP7, in co-transfected cells. Co-expression of USP7 increased the abundance of CRY1 2.6-fold, confirming that USP7 stabilizes CRY1 (Fig 5A, n = 4 biological replicates, *P* = 0.001). We then tested whether USP7 can deubiquitinate CRY1. We co-transfected FLAG-CRY1, USP7 and HA-ubiquitin, immunoprecipitated CRY1, and detected ubiquitinated CRY1 by immunoblotting with anti-HA antibodies. CRY1 is ubiquitinated in this assay (Fig 5B, CRY1^ub^ smear in lane 2), and co-expression of USP7 reduced CRY1 ubiquitination (ubiquitin smear in lane 3 compared to lane 2). Co-expression of MAGEL2 increased CRY1 ubiquitination (Fig 5C, compare CRY1^ub^ smear in lane 3 to lane 2), consistent with the observation that MAGEL2 destabilizes CRY1 (Fig 4). In contrast, co-expression of MAGEL2p.R1187C and MAGEL2p.LL1031AA carrying MHD mutations did not increase CRY1 ubiquitination (Fig 5C, compare lane 3 to lanes 4 and 5). With co-expression of MAGEL2, USP7, and CRY1, the stabilizing effect of USP7 appeared to dominate over the destabilizing effect of MAGEL2 (Fig 5D). Overall, it appears that MAGEL2, USP7, and SCF-E3 ligase complexes can all regulate CRY1 stability and that this process depends on the integrity of the MAGE homology domain.

## Discussion

Protein truncating mutations in *MAGEL2* cause Schaaf-Yang syndrome, while loss of *MAGEL2* and other contiguous genes causes Prader-Willi syndrome. Children with PWS have circadian rhythm disruptions and sleep problems such as nighttime wakening and excessive daytime sleepiness. To date, there are no studies on circadian rhythm and sleep disruptions in individuals with Schaaf-Yang syndrome or individuals with USP7 mutations, due to small sample size and recent clinical discovery. *Magel2* mutant mice exhibit circadian rhythm defects, having a reduced amplitude of activity, increased daytime activity, and fragmented nighttime activity as measured by recording running-wheel activity. *Magel2* mRNA localizes to neurons in the dorsal suprachiasmatic nucleus that express vasopressin, a neuropeptide that is important for mediating circadian rhythm output. *Magel2* mRNA exhibits circadian expression, with peak expression in the late day and rhythmic expression in the suprachiasmatic nuclei of the hypothalamus even in constant darkness [11]. Magel2 is a clock-controlled gene whose expression is disrupted in mice carrying a mutation in the Clock gene. However, the role MAGEL2 plays in regulating circadian rhythm at the cellular level remains to be fully elucidated.

MAGE proteins regulate ubiquitination through their interaction with RING E3 ubiquitin ligases and deubiquitinases [28]. Skp1-Cullin-Fbox E3 ligase complexes target CRY for ubiquitination and degradation, while the MAGEL2-interacting protein USP7 deubiquitinates and stabilizes CRY. The RING finger-like domain-containing protein RBX1 is also in proximity to MAGEL2, and this proximity is disrupted by mutations in the MHD (p.R1187C and p.LL1031AA). This finding is consistent with previous studies showing that mutation of the equivalent dileucine motif in the winged helix A motif of the MHD of MAGEG1/NSMCE3 (NSMCE3p.LL96AA) disrupts its binding to the RING-containing protein NSE1 [28], and MAGEC2 carrying a mutation of the equivalent dileucine motif (MAGEC2p.LL152AA) can no longer bind the RING domain E3 ligase TRIM28. Furthermore, both MAGEL2 MHD mutations suppressed the MAGEL2-induced stabilization of the E3 ligase RNF41 and negated the ability of MAGEL2 to increase trafficking of leptin receptor to the cell surface [15].

**Fig 6:**
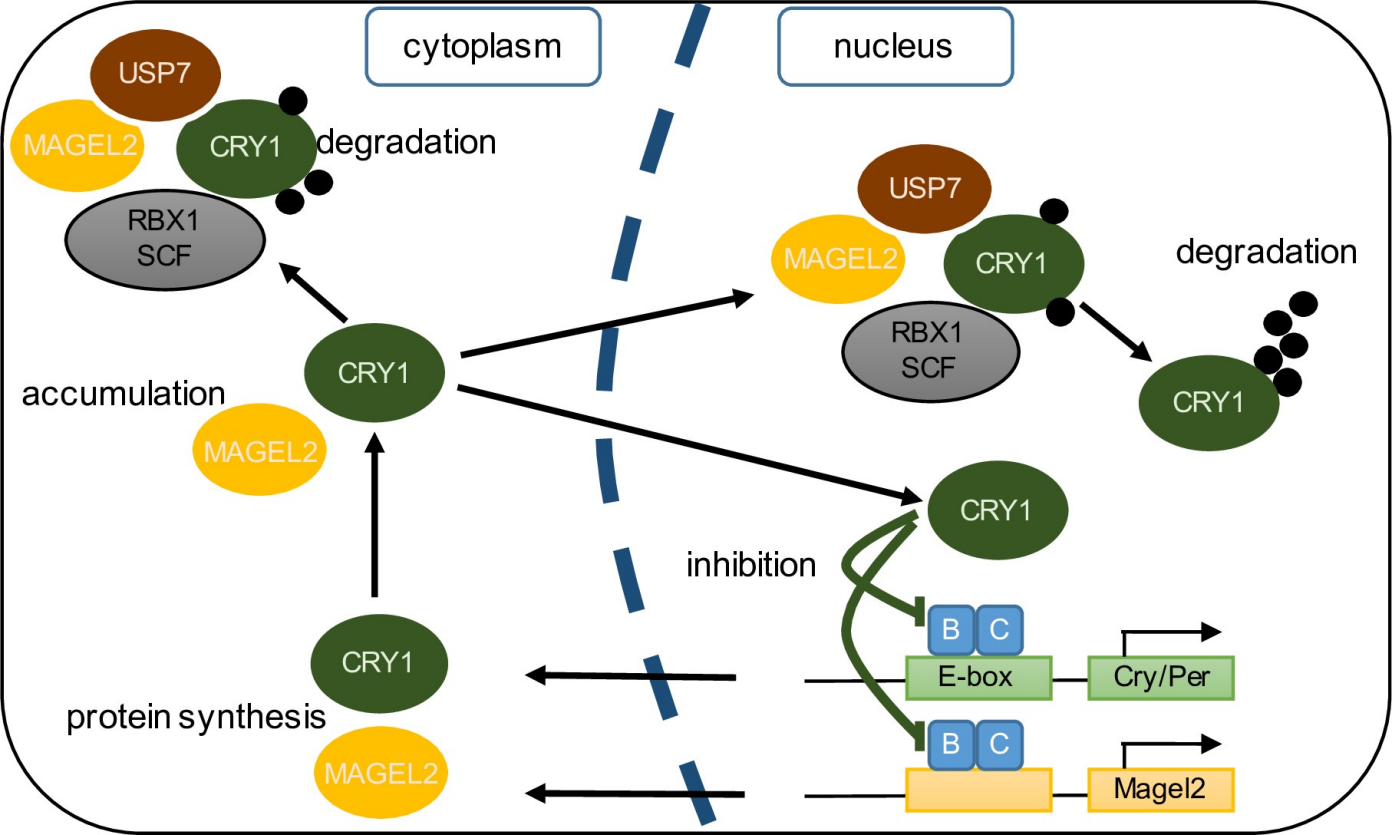
Simplified model for the role of MAGEL2 in circadian rhythm. CLOCK and BMAL1 activate clock-controlled genes, including *MAGEL2*, *CRY1/2* and *PER1/2/3* by binding to elements, such as E-box motifs, in their promoters. Along with PER proteins (not shown), CRY proteins take part in a feedback loop by inhibiting their own expression through repression of CLOCK/BMAL1 activity. CRY1 is modified post-translationally by ubiquitination (black circles). A complex consisting of a RING finger protein (RBX1) and a SKP-Cullin-F-Box complex facilitates ubiquitination of CRY1, while deubiquitinases, including USP7, deubiquitinate CRY1. The balance between ubiquitination and deubiquitination, and between stability and proteasomal degradation, is regulated by various regulatory proteins including the MAGE protein MAGEL2. Translocation between the cytoplasm and the nucleus, and other post-translational modifications such as phosphorylation also modulate the rate of degradation of CRY1 protein.

Given that MAGEL2 interacts with USP7, and that CRY proteins are deubiquitinated and stabilized by USP7, we tested the effect of MAGEL2 and USP7 on the ubiquitination of CRY1. CRY1 ubiquitination decreased in the presence of USP7, and CRY1 protein levels increased (Figs 5A and 6B), as expected. We next considered whether MAGEL2 could be part of a (de)ubiquitination complex that has CRY1 as a substrate. Supporting this idea, MAGEL2 interacts with USP7, and is also proximate to the ubiquitination substrates CRY1 and CRY2 (Fig 2). MAGEL2 co-expression reduced CRY1 protein levels (Fig 4A and 4B) and increased CRY1 ubiquitination (Fig 5). In contrast, co-expression of MHD mutant MAGEL2 protein did not alter CRY1 protein levels or ubiquitination, although the MHD mutant MAGEL2 proteins were still in the vicinity of CRY1. This suggests that the MHD is required for ubiquitin-mediated processes through interactions between the MHD and ubiquitination machinery, but the MHD is not directly required for substrate proximity. Notably, MAGE proteins do not bind the RING domain of E3 ligases, but instead they bind to other parts of RING-containing proteins. Further studies are required to determine whether the interaction between MAGEL2 and RBX1 is sensitive to the exact composition of the SCF complexes [6, 29]. One drawback to our study is the use of heterologous expression in cultured cells to investigate relationships among proteins regulating circadian rhythm. Further validation, *in vivo*, of the results from our study would provide support for the role of MAGEL2 in the regulation of CRY1 protein stability. For example, there may be a compensatory mechanism acting in the mouse brain giving rise to a result that Cry1 levels do not seem to be affected by the loss of *Magel2* despite evidence that MAGEL2 affects CRY1 levels in cell culture.

At the light to dark transition in mice, Clock:Bmal1 protein levels are at their highest in order to initiate transcription of clock-controlled genes during the subjective day. Cry1 is ubiquitinated and degraded at the beginning of the dark period when Per:Cry–mediated repression of Clock:Bmal1 is no longer needed [30–32]. *Magel2* is highly expressed in the suprachiasmatic nucleus of the hypothalamus. Expression is circadian, with highest mRNA levels towards the end of the light period, and about three hours before the peak of *Cry1* expression. It will be important to measure the delay between Magel2 mRNA and protein, once appropriate anti-Magel2 antibodies are generated, to determine whether peak Magel2 protein levels also precede peak Cry1 protein levels. MAGEL2, together with E3 ubiquitin ligase-deubiquitinase complexes, may fine-tune the levels of CRY1 during the circadian cycle, through a ubiquitin-mediated degradation pathway (model, Fig 6). Moreover, we previously showed that MAGEL2 interacts with BMAL1 and PER2 by co-immunoprecipitation, suggesting that MAGEL2 could regulate the ubiquitination and stability of several core circadian rhythm proteins [16]. *Magel2* itself is a clock-controlled gene, forming its own circadian feedback loop with its encoded protein regulating the post-translational modification/ubiquitination of circadian proteins. Notably, even small perturbations to the half-life of circadian proteins can produce overt sleep-wake cycle deficits in animals [7]. In summary, mutations in MAGEL2 (in Schaaf-Yang syndrome) or loss of MAGEL2 function (in PWS) could result in the circadian rhythm and sleep disruptions through disruption of the ubiquitin-dependent feedback loops that regulate circadian rhythm.

## Materials and methods

### Expression analysis in public databases

Images of an adult (56 day old) male C57BL/6J mouse brain, showing Magel2 expression was downloaded from the Allen Brain Atlas [20]. Under the mouse brain tab, in the *in situ* hybridization database, we searched for the expression of *Magel2* in the coronal plane (probe name RP_071204_04_C02). This image is pseudocolored with cells that have the highest level of *Magel2* gene expression in red, yellow indicates moderate expression, and blue indicates low expression. The Circadian Expression Profiles Database (CircaDB [21]) maps the expression of mammalian clock-controlled genes using Genechip 3.2 hybridization data (Affymetrix, Santa Clara, CA). CircaDB was used to identify the expression profile of *Magel2* in the suprachiasmatic nucleus of the hypothalamus in C57BL/6J mice (Probeset #92681_at) and *Clock* delta 19 (C.B6-Clock^m1Jt^/J, JAX stock 016175 [33]) mutant mice (Probeset #gnf1m13016_a_at for *Magel2*, gnf1m10754_at for Cry1) [22]. An estimate of the phase of expression used a Lomb Scargle algorithm [34] and was performed within CircaDB.

### Plasmids

Entry clones with cDNAs for human RBX1, TRIM27, USP7, CRY1, and CRY2 were obtained from the DNASU plasmid repository [35]. Plasmids encoding GFP-WASH1 and GFP-VPS35 were generously provided by Dr. M. Seaman (Cambridge Institute for Medical Research). Mutant MAGEL2 constructs were generated using site-directed mutagenesis to create MAGEL2p.R1187C and MAGEL2p.LL1031AA, as previously described [15]. pDEST-pcDNA5-FLAG, pDEST-pcDNA5-BirA*-FLAG, and pDEST-pcDNA5-BirA*-FLAG-NLS were generously provided by Dr. A-C. Gingras (Lunenfeld-Tanenbaum Research Institute) [36]. Entry clones were recombined into pDEST destination vectors (pcDNA-DEST47 vector for GFP, pcDNA3.1nV5-DEST for V5, pDEST-pcDNA5-FLAG for FLAG, or pDEST-pcDNA5-BirA*-FLAG for BirA*-FLAG) to create epitope-tagged cDNA constructs for expression in mammalian cells. HA-Ub (hemagglutinin-ubiquitin) was expressed from pRK5-HA-Ubiquitin-WT, a gift from Ted Dawson (Addgene plasmid # 17608 [37]).

### Cell lines and transfections

Tissue culture reagents were from Life Technologies (Carlsbad, CA) unless otherwise stated. Human osteosarcoma cells (U2OS, line HTB-96 obtained from ATCC) and human embryonic kidney cells (HEK293T, line HEK293T/17 CRL-11268 from ATCC) were grown in Dulbecco’s modified Eagle medium supplemented with 10% fetal bovine serum, 1% l-glutamine, and 1% penicillin/streptomycin, and cultured at 37°C, in 5% CO_2_. U2OS cells were seeded at a density of 2.3x10^5^ cells/well in a 6-well plate, 24 h before transfection of plasmids using Effectene (Qiagen, Mississauga, Canada). HEK293T cells were seeded at a density of 8.3x10^5^ cells/well in 100 mm dishes, 24 h before transfection of plasmids with Fugene6 (Promega, Madison, WI) at a ratio of 3:1 (Fugene6: DNA). Empty vector plasmids were used as controls and to normalize the amount of plasmid DNA used in each transfection. Stable cell lines carrying tetracycline inducible BirA*-FLAG-MAGEL2 or tetracycline inducible FLAG-MAGEL2 were created in HEK 293 T-Rex Flp-In cells as previously described [15]. MAGEL2 expression was induced by treating cells with 1 μg/ml tetracycline for 24 hours. Control cells were not treated with tetracycline and have low to undetectable levels of MAGEL2 expression. To measure CRY1 protein half-life, cycloheximide (50 μg/ml, C4859 from Sigma) was added to stably transfected HEK cells induced (or not) to express FLAG-MAGEL2, then cell lysates were collected for protein analyses at fixed time points after cycloheximide addition. CRY1 half-life was calculated using the equation of the line fitted as an exponential decay curve (experiment performed in triplicate).

### Immunoblotting

Cell lysates were collected 24 h after transfection in 2x modified sample buffer (20% glycerol, 4% SDS, 2% beta-mercaptoethanol, 1% bromophenol blue, 130 mM Tris-HCl, pH 6.8) with Complete Mini Protease Inhibitor (Roche Applied Science, Indianapolis, IN), sonicated, heated to 65°C, spun at 20 800 x g for 10 min, and boiled for 5 min. Protein was quantified using a BCA protein assay (Pierce, Rockford IL), equal amounts of protein were loaded into each lane, resolved on 10% SDS-PAGE gels, transferred to PVDF membranes and immunoblotted. Signals on immunoblots were visualized on a Kodak imager and signal intensities measured by NIH ImageJ. For nuclear-cytoplasmic fractionation, cells were treated in a two minute fractionation protocol [38]. Briefly, after retaining an aliquot for a whole cell sample, cells were triturated with a P1000 micropipette in ice-cold 0.1% NP40 in PBS, spun briefly, and the supernatant reserved as “cytoplasmic”. The pellet was washed once in ice-cold 0.1%NP40 in PBS, spun briefly, and the pellet “nuclear” resuspended in sample buffer. Whole cell lysates, and cytoplasmic and nuclear samples were then processed for immunoblotting as described for transfected cells. Efficiency of fractionation was confirmed by immunoblotting for TFIID (nuclear protein) and alpha tubulin (cytoplasmic fraction). Blots were re-probed with anti-beta-actin to verify protein loaded into each lane. Co-immunoprecipitation was performed on lysates from a stable cell line carrying a tetracycline inducible FLAG-MAGEL2 construct (lysis buffer 25 mM Tris-Cl, 150 mM NaCl, 1% IGEPAL, 5% glycerol pH 7.4) using the protocol supplied with anti-FLAG M2 magnetic agarose beads (Pierce Anti-DYKDDDDK Magnetic Agarose cat# A36797). Primary antibodies used were: rabbit polyclonal anti-Cryptochrome1 (Abcam ab104736, 1:2000), rabbit polyclonal anti-USP7 (Abcam ab4080, 1:1000), rabbit polyclonal anti-HA (Santa Cruz Biotechnology sc-805, 1:500), mouse monoclonal anti-HA (ThermoFisher Cat #26183, 1:500), mouse monoclonal anti-V5 (Abcam ab27671, 1:1000), rabbit polyclonal anti-FLAG (Sigma #F7425, 1:5000), mouse monoclonal anti-TFIID (Santa Cruz sc-374035, 1:1000), mouse monoclonal anti-β-Tubulin (Santa Cruz sc-5274, 1:1000), chicken polyclonal anti-GFP (Abcam ab13970, 1:5000), or HRP-conjugated mouse monoclonal anti-beta actin antibodies (Sigma #A3854, 1:50,000). Blots were blocked in TBS-TM (5% non-fat dry milk powder in 137 mM NaCl, 0.1% Tween-20, 20 mM Tris-HCl, pH 7.5) for 1 hour at room temperature, then incubated in primary antibodies overnight at 4°C. Blots were washed 3 x 10 min in TBST (137 mM NaCl, 0.1% Tween-20, 20 mM Tris-HCl, pH 7.5) and incubated in secondary antibody for 1 h at room temperature. Secondary antibodies used were: HRP-linked donkey anti-rabbit IgG (Amersham Pharmacia Biotech #NA934, 1:5000), HRP-linked sheep anti-mouse IgG (Amersham Pharmacia Biotech #RPN4201, 1:5000), HRP-linked donkey anti-chicken IgG (Invitrogen #SA1-300, 1:1000). Antibodies were prepared in TBS-TM.

### Mouse brain tissues

All animal studies were conducted in accordance with the Canadian Council on Animal Care Guidelines and Policies with approval from the Animal Care and Use Committee: Health Sciences for the University of Alberta. Adult Magel2^+m/-p^ (Magel2-null) and control littermate Magel2^+m/+p^ mice (Jackson Laboratories C57BL/6-Magel2^tm1Stw/J^, stock 009062) were genotyped by PCR of spare tissue. Postnatal day 10 mouse pups were euthanized and brains removed then the cortex and hypothalamus were dissected and snap frozen in liquid nitrogen. The tissues were ground using pre-chilled disposable plastic pestles and resuspended in 500 μl of 2X modified sample buffer with Complete Mini Protease Inhibitor. The tissues were sonicated on ice (3x for 5 s each with 5 s pauses), incubated at 65°C for 5 min, and centrifuged 10 min at 20000 g at room temperature, and supernatants retained. For immunoblot analysis, 20 μg of protein was subjected to SDS-PAGE and immunoblotting as described above, then blots were probed with rabbit polyclonal anti-Cryptochrome1 (Abcam ab104736, 1:2000) followed by HRP-linked donkey anti-rabbit IgG (Amersham Pharmacia Biotech #NA934, 1:5000).

### BioID proximity labelling interaction assay

BioID experiments were performed essentially as described [15, 39]. U2OS cells were plated at a density of 3x10^5^ cells/well and co-transfected with 0.8 μg of a BirA-expressing plasmid and a plasmid encoding a potentially interacting protein, with 2 wells per co-transfection. After transfection, biotin was added to a final concentration of 50 μM. Cells were processed for BioID 24 h after transfection. A 50 μl aliquot was reserved as “input”. Biotinylated proteins were captured using streptavidin agarose affinity purification and analyzed by immunoblotting as described above.

### Immunofluorescence

U2OS cells were grown and transfected on coverslips in 6 well plates then fixed 24 h later in 4% PFA for 15 min. Cells were blocked in 5% bovine serum albumin in PBSX (PBS, 0.05% Triton X-100) for 15 min, incubated for 1 h at room temperature in primary antibodies rabbit polyclonal anti-FLAG (Sigma #F7425, 1:1000) or mouse monoclonal anti-V5 (Abcam #ab27671, 1:1000)) prepared in 5% bovine serum albumin in PBSX (PBS, 0.05% Triton X-100), and washed in PBSX at 3 x 5 min. Cells were incubated for 1 h at room temperature in secondary antibodies (Alexa Fluor 488 goat anti-rabbit IgG (Life Technologies #A11034, 1:1000) or Alexa Fluor 594 goat anti-mouse IgG (Life Technologies #A11005, 1:1000) prepared in PBSX with 1% normal goat serum. Nuclei were counter-stained with Hoechst 33342 for 15 min (Life Technologies, 1:20,000). Coverslips were mounted onto glass slides using ProLong Gold Antifade Mountant (ThermoFisher #P36930) and sealed using clear nail polish. Transfected cells on coverslips were analyzed using Z-stack imaging taken on a Zeiss LSM 700 confocal microscope with a 40x or 63x oil immersion lens (N. A. 1.4 oil). Visualization of signal intensity and quantification of co-localization were performed in NIH ImageJ using Linescan and EzColocalization Plugins respectively.

### Ubiquitination assay

HEK293T cells were grown and transfected in 100 mm dishes. At 24 h after transfection, cells were incubated overnight with 5 μM MG132 and 25 μM chloroquine in serum-free OPTIMEM media. Cells were washed with PBS and rocked for 30 min in lysis buffer (2% SDS, 150 mM NaCl, 1 mM sodium orthovandate, 1 mM sodium fluoride, 20 mM β-glycerophosphate, 10 mM N-ethylmaleimide, 10 mM Tris-HCl, pH 7.4) with Complete Mini Protease Inhibitor at room temperature. Cell lysates were sonicated, boiled for 10 min, and 50 μl of sample (25% of total sample) was reserved as “input”. The remaining samples were then diluted (150 mM NaCl, 2 mM EDTA, 1% Triton X-100, 10 mM Tris-HCl, pH 8) and incubated on a rocker at 4°C for 1 h. Samples were centrifuged at 20000 g for 30 min then precleared with 20 μl Sepharose 4B beads (Sigma #4B200) at 4°C for 1 h on a rocker. Precleared samples were centrifuged at 3000 g for 2 min and the supernatant was transferred to a new tube. Lysates were incubated overnight at 4°C with anti-FLAG M2 Affinity gel (Sigma #A2220) on a rocker. Samples were centrifuged at 3000 g for 2 min and washed twice with wash buffer (1 M NaCl, 1 mM EDTA, 1% IGEPAL, 10 mM Tris-HCl, pH 8). Beads were resuspended in collection buffer (3% SDS, 10% glycerol, 62.5 mM Tris-HCl, pH 8) then used for immunoblot analysis as described above.

### Statistics

A Fisher exact test was used to determine the effect of co-expression of MAGEL2 on the subcellular localization of CRY1, with *P*<0.05 deemed to be statistically significant. Student t-test was used to test whether there were differences between groups of triplicate or quadruplicate samples, with *P*<0.05 used as a standard for statistical significance.

## Supporting information

S1 Fig Expression of Magel2 is high in the suprachiasmatic nucleus of the hypothalamus and follows a circadian pattern. A) RNA *in situ* hybridization on an adult mouse brain section in the coronal plane, demonstrating high expression of *Magel2* in the hypothalamus (dark blue/purple signal). A’) inset shows expression in the suprachiasmatic nucleus (pseudocolored, red signal is highest expression; yellow signal is moderate expression). Data from Allen Brain Atlas. B) Expression of murine *Magel2* follows a highly circadian pattern in the suprachiasmatic nucleus of the hypothalamus. C) Expression of *Magel2* in WT mice (orange curve) and in mice carrying a *Clock* gene mutation (blue curve), over a time period of ~48 hours including light (shaded white) and dark (shaded gray) periods. D) Expression of *Cry1* in WT mice (orange curve) and in mice carrying a *Clock* gene mutation (blue curve). Phase of expression in the wild-type mice is indicated. B-D, Data from Circadian Expression Profiles Database, CircaDB, in the suprachiasmatic nucleus of the hypothalamus.
(TIF)

S2 Fig Abundance of CRY1 in cells expressing MAGEL2. A) Whole cell lysates (W) from transfected U2OS cells were fractionated into nuclear (N) and cytoplasmic (C) fractions, and recombinant proteins in these samples were detected and quantified by immunoblotting. The quality of the fractionation procedure was tested by immunoblotting the same samples for an endogenous nuclear protein (TFIID) and an endogenous cytoplasmic protein (tubulin). B) Whole cell lysates (W) from HEK293-MAGEL2 cells were fractionated into nuclear (N) and cytoplasmic (C) fractions, and both recombinant FLAG-MAGEL2 and endogenous CRY1 in these samples were detected by immunoblotting. Cells were either from cultures induced (+) or uninduced (-) with tetracycline (tet) to promote expression of FLAG-MAGEL2.
(TIF)

S3 Fig Abundance of Cry1 in mouse hypothalamus and cortex (brain). Protein lysates from dissected regions of the brain from postnatal day 10 mice (Magel2^tm1Stw^ or wildtype littermate) were subjected to SDS-PAGE and immunoblotting, then blots were probed with anti-Cry1 antibodies to detect Cry1 protein (C). Left, lysates from cortex from 4 Magel2 mutant (m) and 4 wildtype (w) mice, and right, lysates from hypothalamus from 4 Magel2 mutant (m) and 3 wildtype (w) mice, and lysate from cultured U2OS cells transiently expressing CRY1-FLAG as a positive control.
(TIF)

S4 Fig The deubiquitinase USP7 is in proximity to MAGEL2 as detected by BioID. U2OS cells were transiently transfected with constructs encoding epitope-tagged proteins, incubated with biotin, and collected 24 h after transfection. A portion of the cell lysate was removed and retained as input. Subsequently, streptavidin affinity purification captured V5-tagged MAGEL2 that was biotinylated by BirA*-USP7 (bound).
(TIF)

S5 Fig Full blots for Figs 1, 3, 4, 5. (PDF)
