# Supplementary figures and images for: A MAGEL2-deubiquitinase complex modulates the ubiquitination of circadian rhythm protein CRY1
Source: PLoS One. 2020 Apr 21;15(4):e0230874. doi: 10.1371/journal.pone.0230874 (PMC7173924; doi:10.1371/journal.pone.0230874)

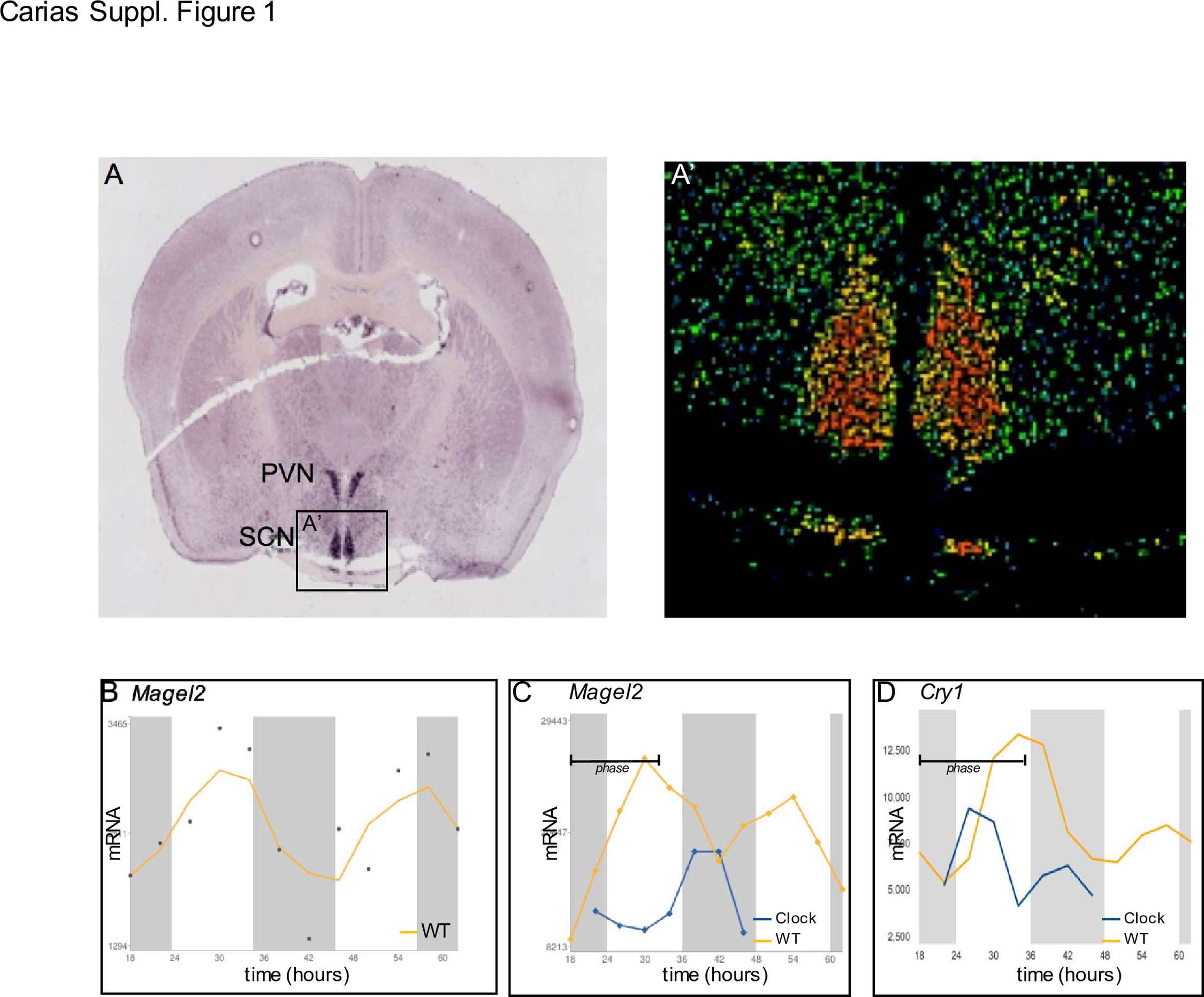

Supplement: S1 Fig — A) RNA in situ hybridization on an adult mouse brain section in the coronal plane, demonstrating high expression of Magel2 in the hypothalamus (dark blue/purple signal). A’) inset shows expression in the suprachiasmatic nucleus (pseudocolored, red signal is highest expression; yellow signal is moderate expression). Data from Allen Brain Atlas. B) Expression of murine Magel2 follows a highly circadian pattern in the suprachiasmatic nucleus of the hypothalamus. C) Expression of Magel2 in WT mice (orange curve) and in mice carrying a Clock gene mutation (blue curve), over a time period of ~48 hours including light (shaded white) and dark (shaded gray) periods. D) Expression of Cry1 in WT mice (orange curve) and in mice carrying a Clock gene mutation (blue curve). Phase of expression in the wild-type mice is indicated. B-D, Data from Circadian Expression Profiles Database, CircaDB, in the suprachiasmatic nucleus of the hypothalamus. (TIF) [file pone.0230874.s001.tif]

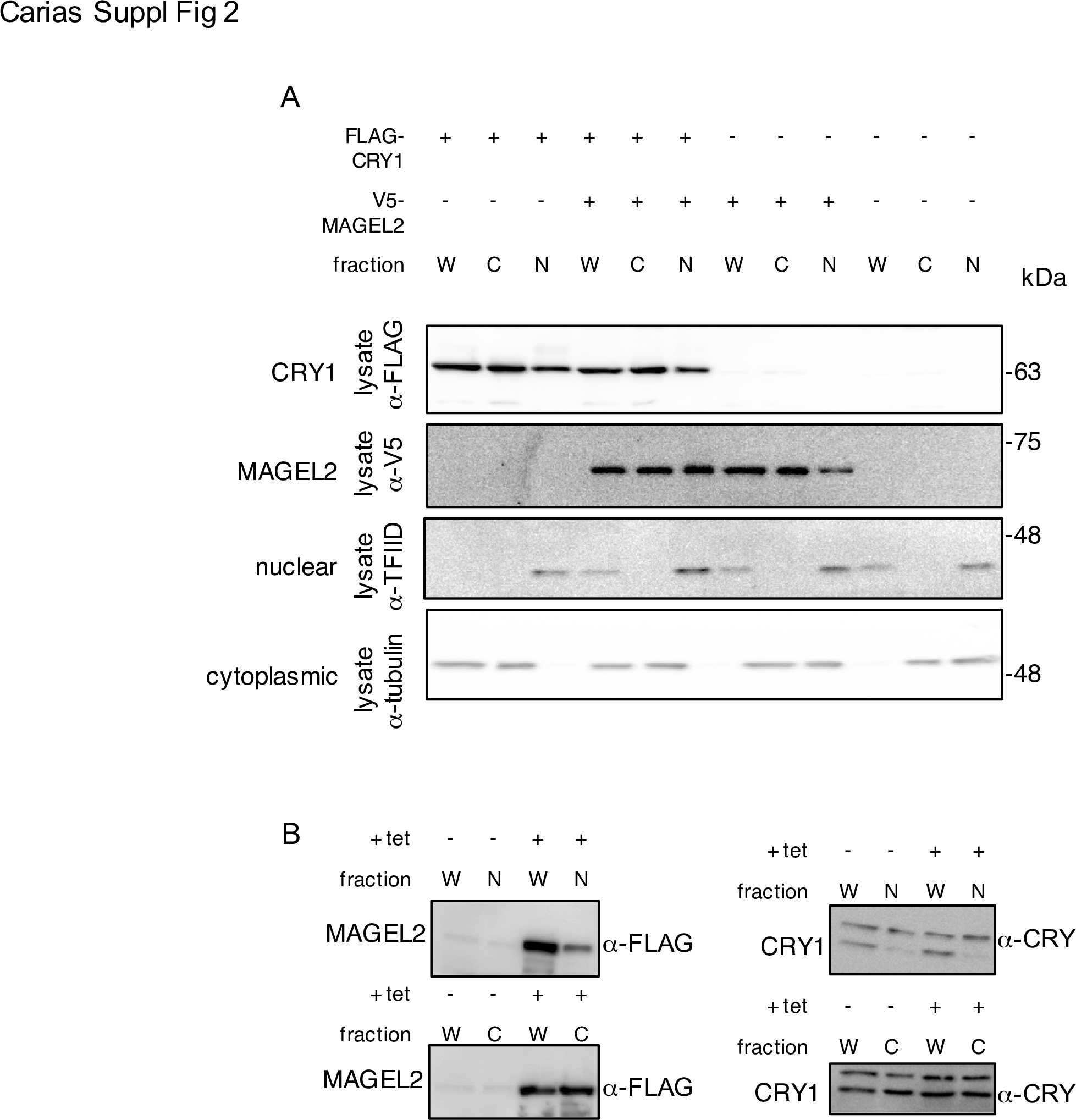

Supplement: S2 Fig — A) Whole cell lysates (W) from transfected U2OS cells were fractionated into nuclear (N) and cytoplasmic (C) fractions, and recombinant proteins in these samples were detected and quantified by immunoblotting. The quality of the fractionation procedure was tested by immunoblotting the same samples for an endogenous nuclear protein (TFIID) and an endogenous cytoplasmic protein (tubulin). B) Whole cell lysates (W) from HEK293-MAGEL2 cells were fractionated into nuclear (N) and cytoplasmic (C) fractions, and both recombinant FLAG-MAGEL2 and endogenous CRY1 in these samples were detected by immunoblotting. Cells were either from cultures induced (+) or uninduced (-) with tetracycline (tet) to promote expression of FLAG-MAGEL2. (TIF) [file pone.0230874.s002.tif]

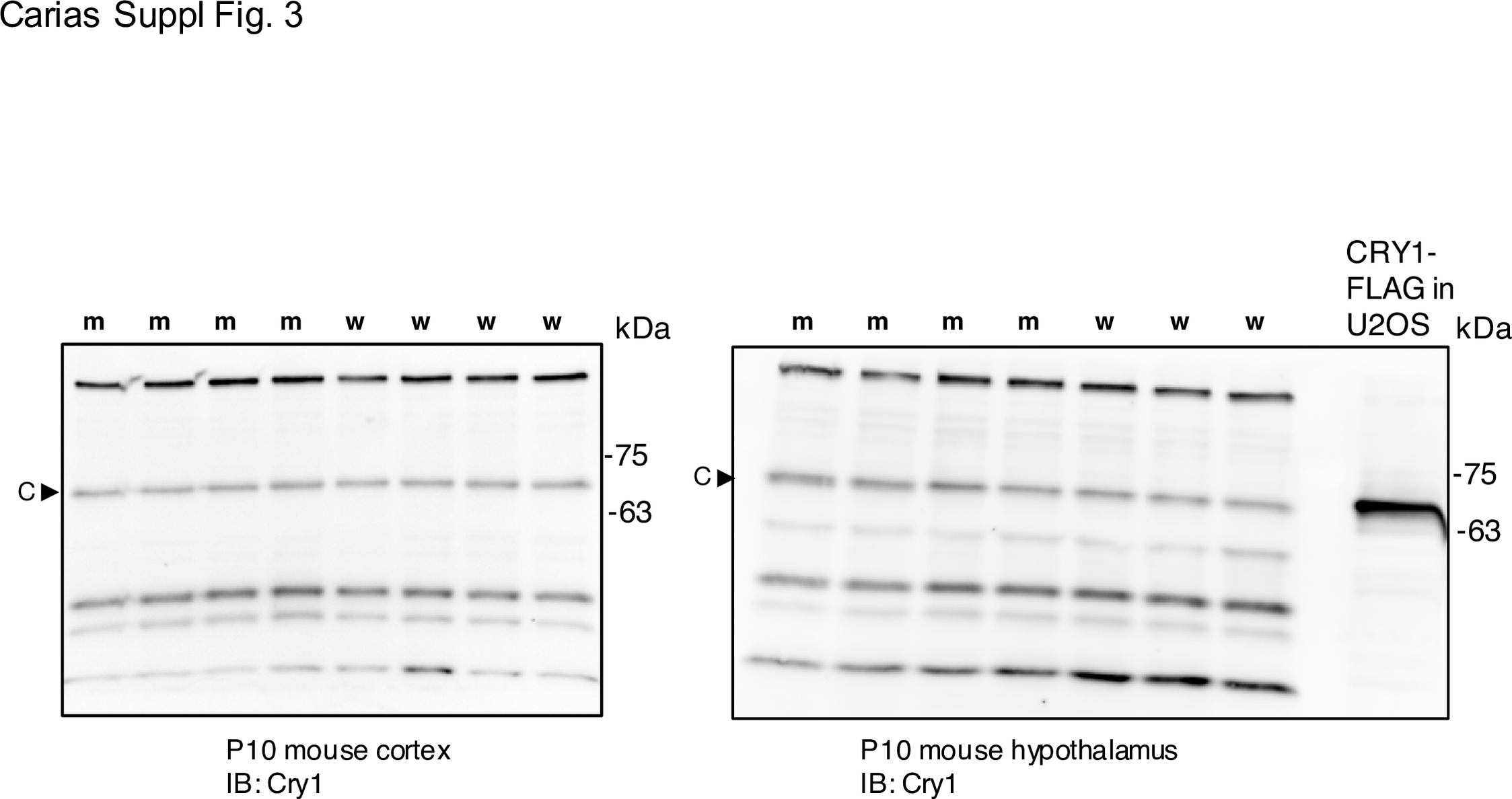

Supplement: S3 Fig — Protein lysates from dissected regions of the brain from postnatal day 10 mice (Magel2tm1Stw or wildtype littermate) were subjected to SDS-PAGE and immunoblotting, then blots were probed with anti-Cry1 antibodies to detect Cry1 protein (C). Left, lysates from cortex from 4 Magel2 mutant (m) and 4 wildtype (w) mice, and right, lysates from hypothalamus from 4 Magel2 mutant (m) and 3 wildtype (w) mice, and lysate from cultured U2OS cells transiently expressing CRY1-FLAG as a positive control. (TIF) [file pone.0230874.s003.tif]

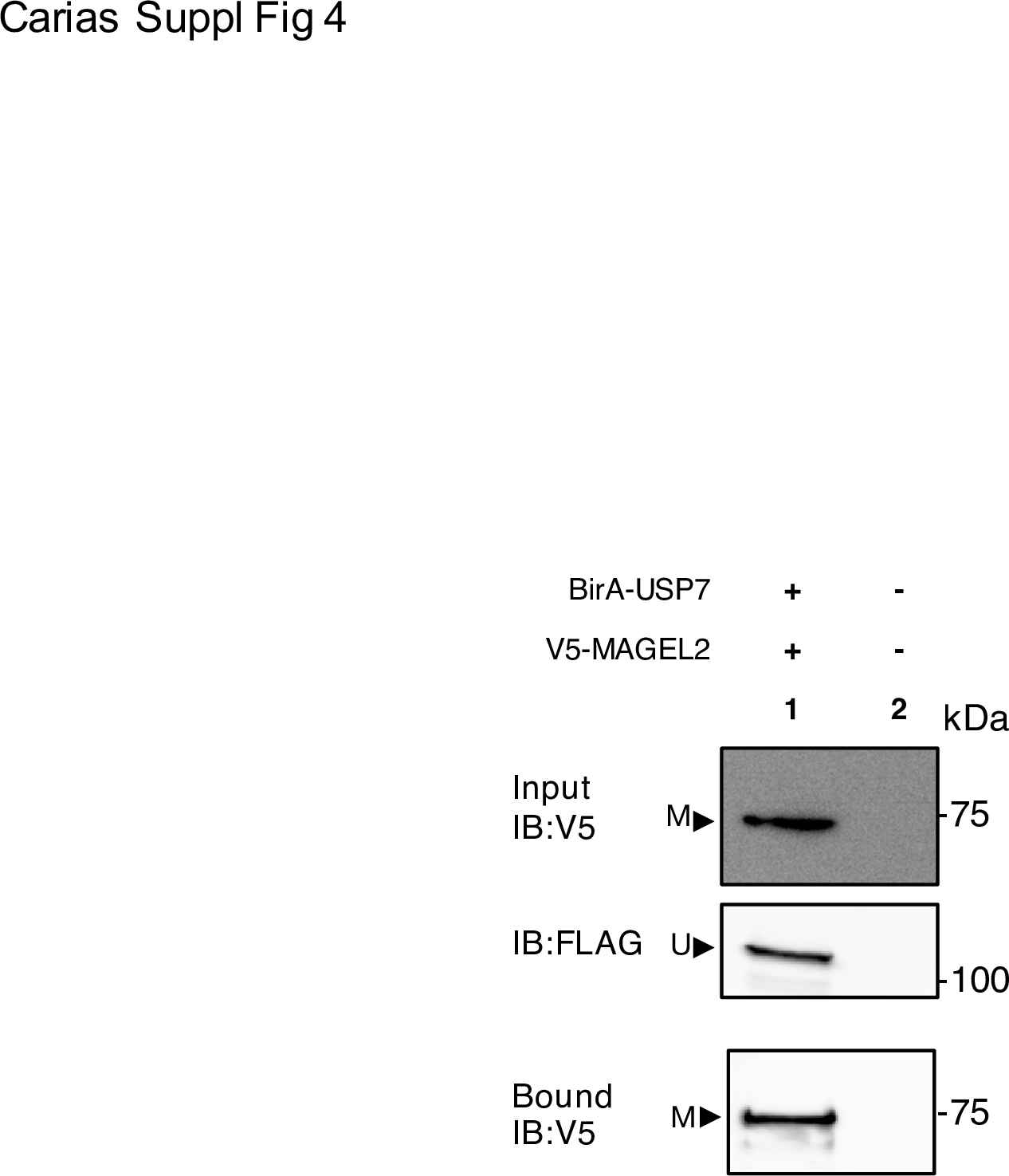

Supplement: S4 Fig — U2OS cells were transiently transfected with constructs encoding epitope-tagged proteins, incubated with biotin, and collected 24 h after transfection. A portion of the cell lysate was removed and retained as input. Subsequently, streptavidin affinity purification captured V5-tagged MAGEL2 that was biotinylated by BirA*-USP7 (bound). (TIF) [file pone.0230874.s004.tif]
