# Supplementary material for: A MAGEL2-deubiquitinase complex modulates the ubiquitination of circadian rhythm protein CRY1
Source: PLoS One. 2020 Apr 21;15(4):e0230874. doi: 10.1371/journal.pone.0230874 (PMC7173924; doi:10.1371/journal.pone.0230874)

Full blots: Figure 1.

A

Anti GFP

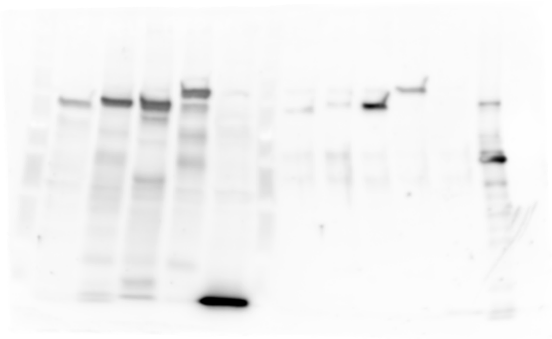

Anti FLAG

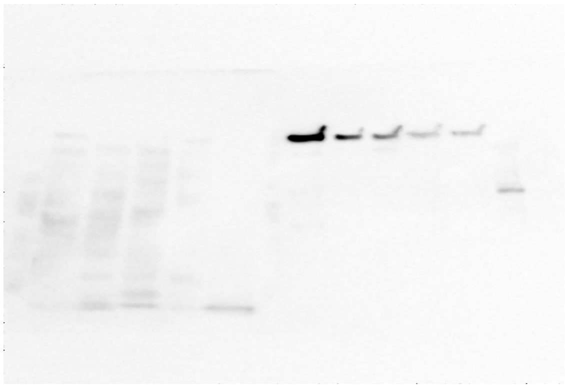

B

Anti GFP

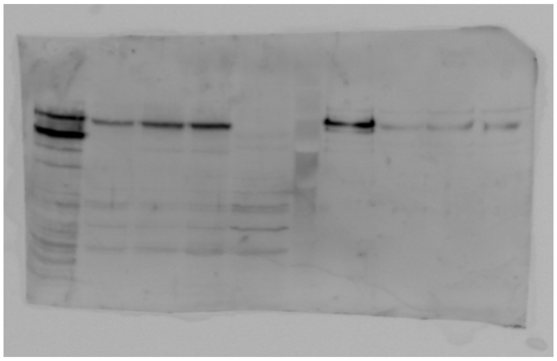

Anti FLAG

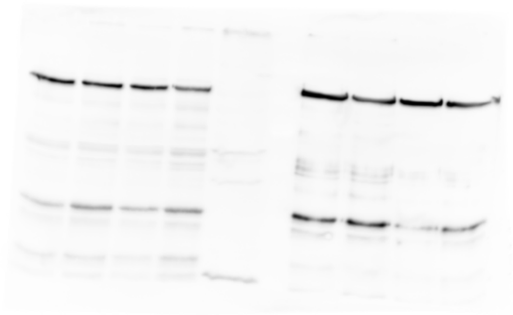

Carias Fig. 3

A

Anti V5

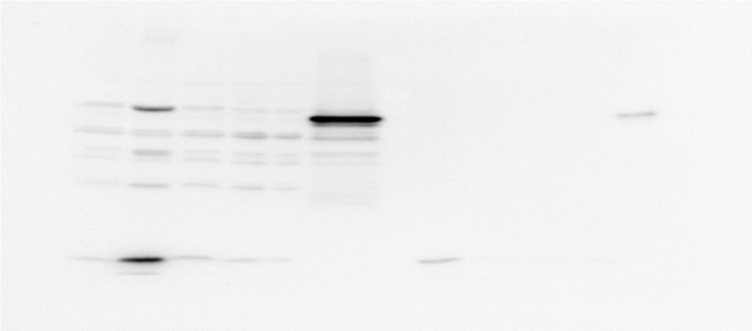

Anti FLAG

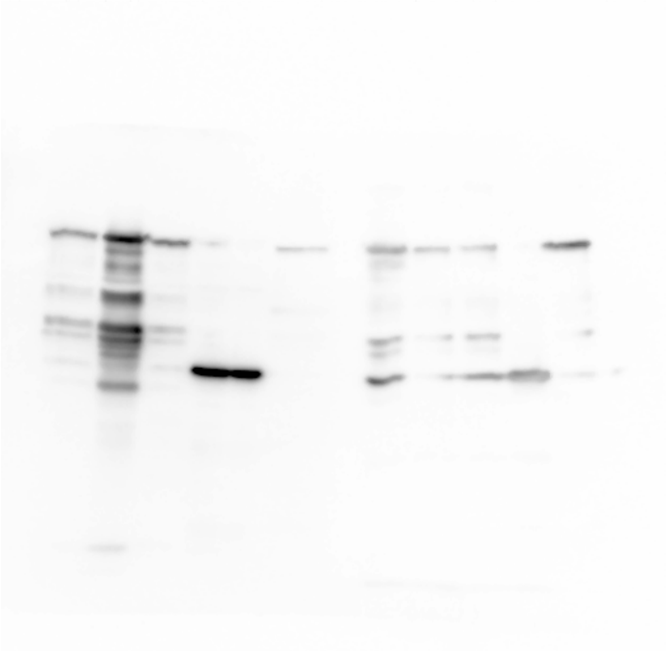

B

Anti V5

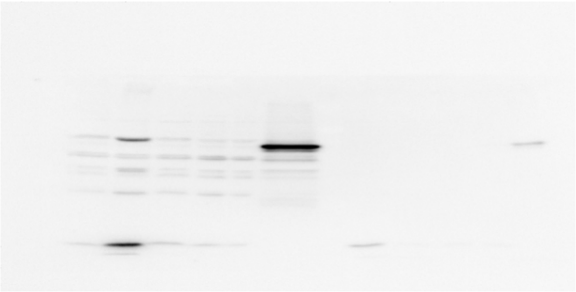

Anti FLAG

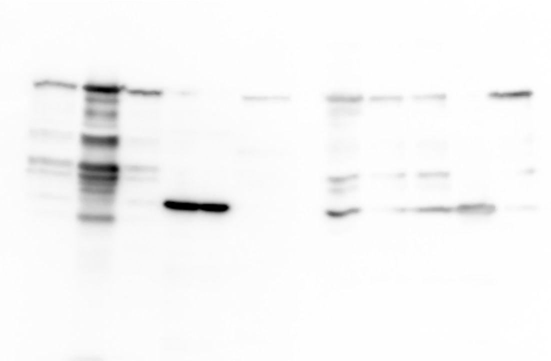

Carias Figure 4

A

Anti FLAG

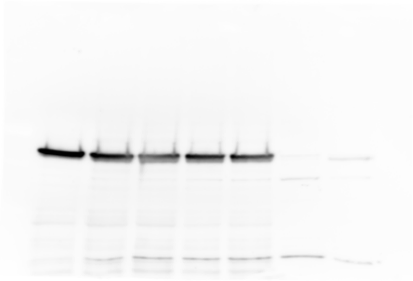

Anti V5

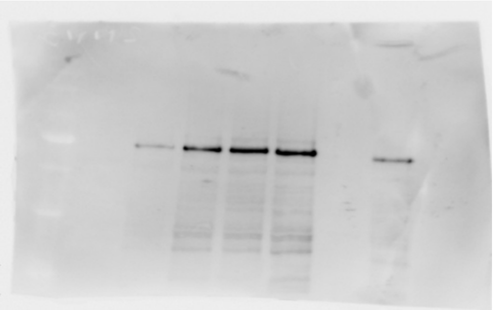

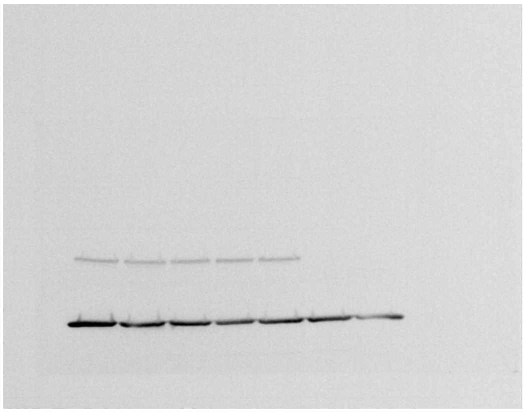

Anti actin

B

Anti FLAG

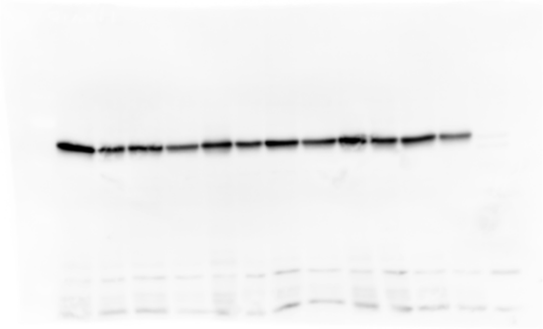

Anti V5

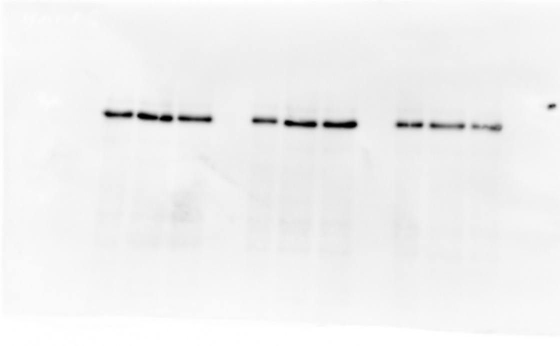

Anti actin

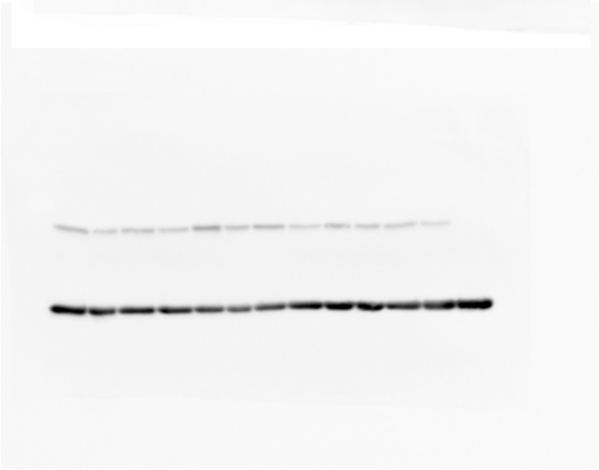

Carias Figure 5

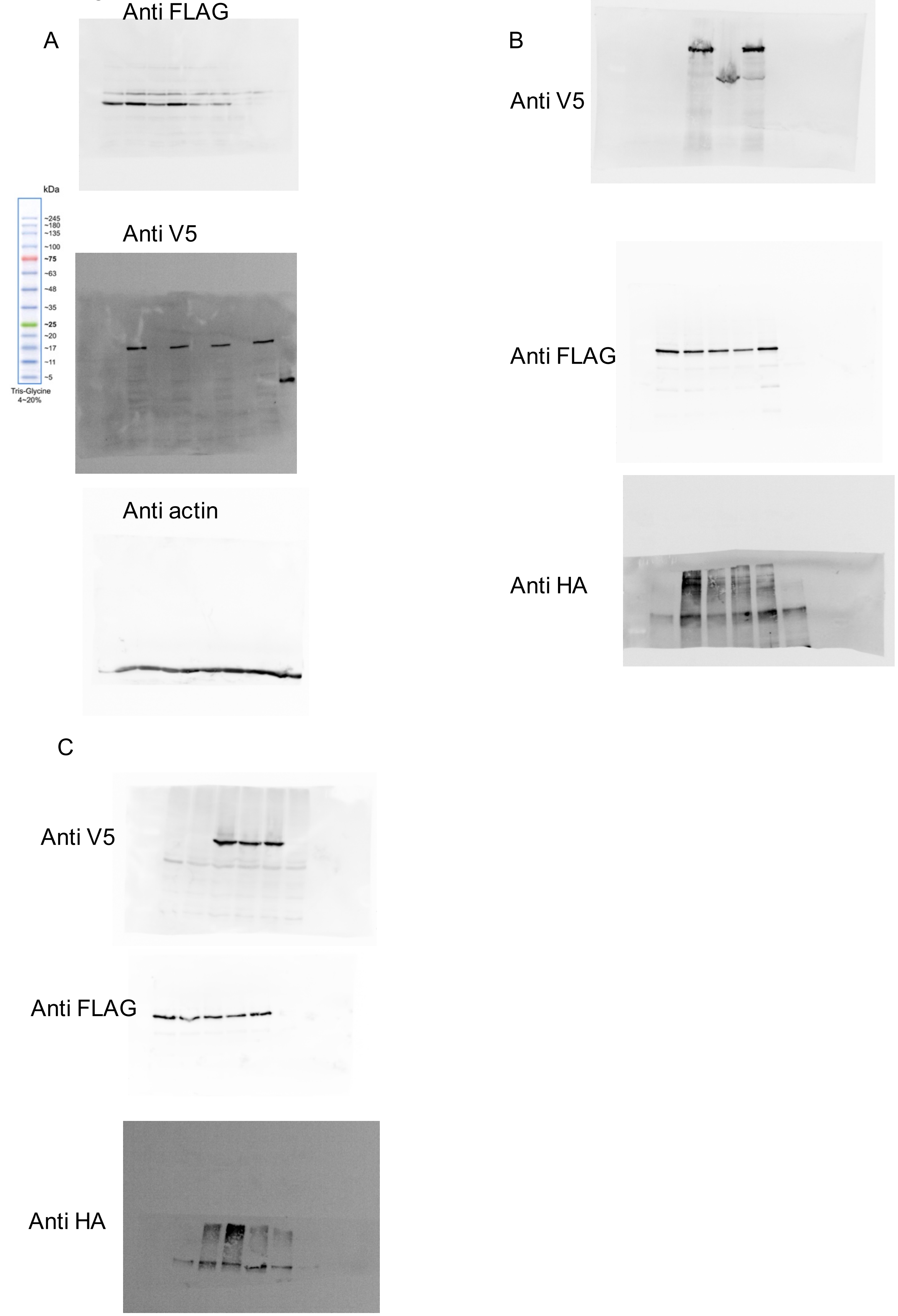

Supplement: S5 Fig — (PDF) [file pone.0230874.s005.pdf]
